# Effect of temperature (cooking and freezing) on the concentration of oxytetracycline residue in experimentally induced birds

**DOI:** 10.14202/vetworld.2018.167-171

**Published:** 2018-02-10

**Authors:** Ezenduka Ekene Vivienne, Okorie-kanu Onyinye Josephine, Nwanta John Anaelom

**Affiliations:** Department of Veterinary Public Health and Preventive Medicine, University of Nigeria, Nsukka, Nigeria

**Keywords:** antimicrobials, cooking methods, drug residue, oxytetracycline

## Abstract

**Aim:**

The objective of this study was to determine the effect of varying temperatures (different cooking methods and freezing) on the concentration of oxytetracycline (OTC) residues in tissues of broiler birds.

**Materials and Methods:**

Fifty, 5-week-old birds were purchased and acclimatized for 3 weeks while being fed antibiotic-free feed and water. Four birds were then tested for residue and in the absence; the remaining birds were injected intramuscularly with oxytetracycline at its therapeutic dose. Muscle and liver samples of the treated birds were harvested and checked for OTC residues before subjecting them to boiling, microwaving, and roasting. The three plate test was used for the residue detection.

**Result:**

OTC was detected at both pH 6.0 and pH 7.2 but not detected at pH 8.0. Roasting and boiling significantly reduced the concentration of oxytetracycline in muscle by 53.6% and 69.6%, respectively, at pH 6.0, microwaving reduced the concentration by 49.1% but was not statistically significant. The same pattern was followed at pH 7.2 with reduction of 34.3%, 53.2%, and 67.7% for microwaved, roasted, and boiled. For the liver tissues, there was a significant reduction in the concentration for both pH: 6.0 (57.75%, 79.75%, and 89%; pH 7.2 (48.06%, 79.6%, and 88.79%) for boiled, microwaved, and roasted samples. Boiling had a greater reduction effect for muscle samples while roasting had a greater reduction in liver samples at both pHs. Freezing at −10°C had no effect on the concentration of OTC even after 9 days.

**Conclusion:**

The significant reduction of OTC concentration by cooking indicates that consumers may not be at risk of the effects of OTC residues in meat, but microwaving meat may not reduce the concentration below the maximum residue limit if the initial concentration is very high. Therefore, routine monitoring of drug residues in farms and abattoirs is still advocated.

## Introduction

Antimicrobials are widely used in livestock, and poultry production for the purposes of prevention and treatment of diseases and growth promotion [1,2] and the tetracyclines (TCs) are ranked among the frequently used [3,4]. These drugs accumulate in tissues and eggs as residues if the withdrawal periods are not observed before slaughter or before egg collection. Residues of these drugs are of public health importance because of the deleterious effect they have in humans. Some of these effects include development of resistant strains of microorganisms, allergy, autoimmunity, and nephrotoxicity which can be particularly caused by gentamicin and distortion of the microflora [5]. Some of the drugs have been banned for use in food animals especially furazolidone and chloramphenicol because of their mutagenic and bone marrow toxicity effect, respectively.

The European four plate test is a reference microbiological method for the detection of antimicrobial residues in foods of animal origin. It is said to be semi-quantitative because it can detect different classes of antimicrobials. As the name implies, it consists four different test plates seeded with *Bacillus subtilis* and *Micrococcus luteus* at different pH levels (pH 6.0, 7.2, and 8.0). pH 6.0 best detects beta-lactams and TCs, pH 7.2 best detects sulfonamides, and pH 8.0 best detects aminoglycosides while pH 8.0 seeded with *M. luteus* best detects macrolides [6,7].

In developed countries, many researchers have been interested in evaluating whether antibiotic residues can be destroyed by cooking procedures, pasteurization, or canning processes [8-12]. Traditionally, heat stability of antibiotics has been studied based on a change in concentration using microbiological test methods. Few studies evaluating the heat stability of veterinary drug residues have been carried out using both microbiological and immunoassay methods [13]. Previous studies have suggested that sulfamethazine, oxacillin, chloramphenicol, aminoglycosides, quinolones, clindamycin, novobiocin, trimethoprim, vancomycin, and azlocillin are heat-stable [9,14,15], while oxytetracycline (OTC) and erythromycin were shown to be heat-labile [11]. On the other hand, several β-lactams such as penicillin G, ampicillin, and amoxicillin appear partially heat-labile [14]. Antibiotics of the same class were also reported to show different heat stability depending on different matrices and heating treatments involved [13,16,17].

Since meat is always heated before consumption, few reports have been published about the effect of heating on the stability of TCs residues in chicken. The fate of drug residues during heat processing is, however, unclear. Although freezing is a form of preservation method of meat by impeding the growth of microorganisms, the fate of antimicrobial residues concentration when frozen with time is unknown. This study will thus investigate the effect of heat treatment and freezing on OTC residues in liver and muscle tissues of broiler chicken using the three plate test (TPT), a microbiological method of detection. The TPT, also a type of plate test was chosen for the study since the focus antimicrobial is OTC belonging to the class of TC and they are best detected with *B. subtilis* at pH 6.0.

## Materials and Methods

### Ethical approval

This study was conducted in accordance with the Ethics and Regulation Guiding the Use of Animals as approved by the University of Nigeria, Nsukka.

### Study design

Fifty, 5-week-old broilers were bought from a major poultry farmer. The birds were fed *ad libitum* with antimicrobial free feed and water for 3 weeks. After the 3 weeks waiting period, 4 birds were slaughtered and the breast muscles and liver were collected as post-slaughter sample matrix. The samples were tested for the presence of antimicrobial residues using TPT. All the samples from the four birds were tested negative for antimicrobial residues.

### Experimental drug administration

In the absence of antimicrobial residues in the birds after the waiting period, the birds were injected intramuscularly at the breast muscle with long-acting OTC (Coophavet, France) at the label therapeutic dose of 20 mg/kg body weight.

### Testing for OTC residue in experimental birds

After 24 h post drug administration, 2 birds were slaughtered daily, the liver and muscle harvested, extracted and tested for residue until no residue was detected in the organs.

### Sample preparation/organ juice extraction

5 g of each organ sample (raw and heat treated) was macerated using sterile pestle and mortar, emulsified with 5 ml of distilled water and centrifuged at 5000 rpm for 10 min as described by Nonga *et al.*, 2009 [18]. The supernatant was decanted into Eppendorf tubes and stored for analysis. All the positive tissues with high concentration of OTC residue were grouped into four parts; the first part was analyzed for the effect of freezing and the remaining three parts for the effect of heat treatment (boiling, microwaving, and roasting).

### Effect of heat treatment

10 g each of all the positive liver and muscle tissues were weighed before heat treatment.

#### Boiling

The weighed samples were placed into a strainer, immersed in 10 ml of water bath preheated to 100°C and cooked for 30 min and allowed to cool before meat juice extraction.

#### Roasting

The weighed samples were placed on a metal baking tray and cooked to well done in an electric oven at 200°C for 30 min and allowed to cool before extraction.

#### Microwaving

The sample portion was placed in the microwave and cooked under full power (900 W) for 3 min, removed, and allowed to cool before extraction. All the heat-treated samples were tested for residue.

### Effect of freezing on OTC residue

A portion of the positive samples were kept frozen at −10°C and tested for residue first after 24 h, and subsequently, every 3 days for 10 days.

### OTC residue testing

This was done using the TPT as described by Heitzman 1994 [6], with *B. subtili*s (BGA), Merck, Darmstadt, and Germany) as the test organism and Mueller-Hinton agar (Oxoid) as the medium. Susceptibility of the organism; *B. subtilis* was tested with commercially produced antibiotic discs of the TCs using Kirby and Bauer disc diffusion methods of determining susceptibility as per recommendation of Clinical Laboratory Standard Institute [19]. Three batches of Mueller-Hinton agar broth were prepared and adjusted to pH 6.0 and 7.2 with dilute sulfuric acid (H_2_SO_4_) and to 8.0 with sodium hydroxide (NaOH). The adjusted media were sterilized and poured into sterile Petri dishes and allowed to gel. Each plate was seeded with *B. subtilis*. 3 wells were bored on each agar plate. 80 µl of the organ extracts (liver and muscle) were inoculated in 2 wells, each well representing an organ, the remaining well was inoculated with 80 µl of distilled water as negative control. The plates were incubated at 30°C for 18-24 h, clear zone of inhibition with annular diameter ≥2 mm indicates positive for OTC [5].

### Statistical analysis

Data from the study were analyzed in GraphPad Prism Statistical software version 5.02 (www.graphpad.com). 2 sample t-test was used to compare the mean values of raw samples and the different cooking methods while analysis of variance (ANOVA) was used to compare the mean values of the freezing times. The alpha value of significance was set at the p level of <0.05.

## Result

The isolate (*B. subtilis*) was susceptible to all the antimicrobials tested including OTC. All the positive samples with diameters above 4 mm were used. This means that the positive samples used were above the maximum residue limit (MRL) of OTC.

Table-1 summarizes the effect of different cooking methods on the concentration of OTC residues in muscles tissue using TPT at pH 6.0. The mean inhibition zones of raw muscles (7 mm) at pH 6.0 were significantly (p≤0.05) reduced in boiled (2.13 mm) and roasted (3.25 mm) samples with 69.57% and 53.57% reductions, respectively. The 49.14% reduction by microwaving (3.56 mm) was not statistically significant.

**Table-1:** Comparison of inhibition zones between raw muscles and different cooking methods at pH 6.0.

Muscle	Mean inhibition zone (mm)	Difference from raw	(%) different	SEM (+ or−)	t-test	df	p
Raw	7.00	-	-	1.45	-	-	-
Microwaved	3.56	3.44	49.14	1.11	1.88	14	0.081
Roasted	3.25	3.75	53.57	0.92	2.00	14	0.047
Boiled	2.13	4.87	69.57	0.76	2.00	14	0.010

SEM=Standard error of the mean

Table-2 summarizes the effect of the cooking methods on OTC residue in muscle at pH 7.2. There was no statistical differences (p>0.05) in the mean values of raw (7 mm) and microwaved (3.94 mm) with 34.33% reduction. Statistical differences were observed between raw versus roasted (2.81 mm) and raw versus boiled (1.94 mm) muscle samples with 53.7% and 67.99%, respectively.

**Table-2:** Comparison of inhibition zones between raw muscles and different cooking methods at pH 7.2.

Muscle	Mean	Difference from raw	(%) different	SEM	t-test	df	p
Raw	6.00	-	-	1.169	-	-	-
Microwaved	3.94	2.06	34.33	1.49	1.09	14	0.290
Roasted	2.81	3.19	53.17	0.80	2.00	14	0.044
Boiled	1.94	4.06	67.66	0.57	3.00	14	0.007

SEM=Standard error of the mean

Table-3 summarizes the effect of different cooking methods on the concentration of OTC residues in liver tissues at pH 6.0. The mean inhibition zone of raw (4.0 mm) liver at pH 6.0 was significantly (p≤0.05) reduced by the three cooking methods at 57.73%, 79.75%, and 89% for boiled, microwaved, and roasted, respectively.

**Table-3:** Comparison of inhibition zones between raw liver and different cooking methods at pH 6.0.

Muscle	Mean	Difference from raw	(%) difference	SEM	t-test	df	p
Raw	4.00	-	-	0.63	-	-	-
Boiled	1.69	2.31	57.75	0.75	2.37	14	0.032
Microwaved	0.81	3.19	79.75	0.35	4.43	14	0.006
Roasted	0.44	3.56	89.00	0.22	5.36	14	0.001

SEM=Standard error of the mean

Table-4 summarizes the effect of cooking methods on OTC residue in liver at pH 7.2. The mean inhibition zone of raw liver was also reduced by all the cooking methods at 48.06%, 79.63%, and 88.79% for boiled, microwaved, and roasted, respectively.

**Table-4:** Comparison of inhibition zones between raw Liver and different cooking methods at pH 7.2.

Muscle	Mean	Difference from raw	(%) difference	SEM	t-test	df	p
Raw	4.91	-	-	0.61	-	-	-
Boiled	2.55	2.36	48.06	0.56	2.85	20	0.009
Microwaved	1.00	3.91	79.63	0.26	5.89	20	<0.001
Roasted	0.55	4.36	88.79	0.64	6.78	20	<0.001

SEM=Standard error of the mean

**Figure-1:**
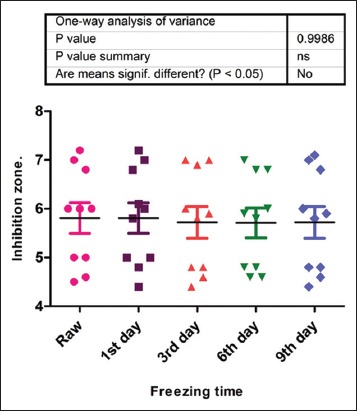
Effect of freezing on the concentration of oxytetracycline residue.

For the effect of freezing time on OTC concentration in muscle and liver tissues at both pH, at pH 6.0, muscle sample with initial mean inhibition zone of 5.81 changed to 5.81, 5.72, and 5.71; at pH 7.2, the initial mean inhibition zone change from 6.0 mm to 6.0, 6.2, and 6.0 at the 3^rd^, 6^th^, and 9^th^ days of freezing, respectively. The same pattern was followed for liver samples. There was little or no reduction in the mean concentrations of freezing in both tissues at different time intervals. The summary of the effect is shown in Figure-1.

## Discussion

The effect of cooking procedures on OTC in poultry was determined by TPT. The TPT, a variant of the four plate test (FPT) best detects the TCs at pH 6.0 with *B. subtilis* as the test organism, but this study also found that the TCs are comfortably detected at pH 7.2 as well. The inhibition zones noted in this work could be assumed to be at or above the violative level of OTC because plate test with *B. subtilis* cannot detect residue when their concentrations are below or around the permissible limit [7,5]. The result for both pHs in muscle gave a net mean inhibitory zone of 34-69% reduction with the statistically significant maximum (69%) reduction shown by boiling and the minimum shown by microwaving. A similar work done by Javadi *et a*l. [20], on doxycycline, had a net decrease in mean inhibitory zones of 33-100% with maximum and minimum inhibitory zone also shown by boiling and microwaving, respectively. Since the effect of an increase in cooking time was not done in this study, it may not be out of place to suggest that microwaving had the lowest decreasing effect on OTC concentration due to short cooking time.

The highest reduction of OTC in muscles by boiling in this study also agrees with the work of Rose *et al*. [17], with observed substantial net reductions in OTC of 35-94% and also in pig muscle with a net reduction of 48-60% [21]. The reduction of OTC in liver samples, on the other hand, had a higher net mean inhibitory reduction for both pHs at 58-89% with roasting having the highest reduction and boiling the least. Although Javadi *et al*. [20] had total (100%) reduction of inhibitory zones for both muscle and liver samples at pH 6.0 and 7.2, the mean inhibitory zones of the raw samples were <2 mm in diameter, and so a complete clearance of residues was achieved. In this study, however, the least mean value of the samples was 4 mm; therefore, there was no 100% clearance, this means that very high concentrations of OTC residues may not be reduced below the MRL by heating. Al-Ghamd *et al*. [22] also did not achieve complete clearance of OTC due to the initial high concentration of the raw samples.

The results of this study are consistent with several other studies that reported a decrease in antimicrobial residues concentrations in foods following heat treatment. Therefore, the possibility of reducing the apparent toxic effect of these drugs to consumers is increased. Reduction in TC concentrations during boiling was due to the migration of the TC from the meat to cooking medium (water) [9], while during the microwaving and roasting processes, reduction was due to juice exuding out from the meat. The overall loss of TC residues was due to denaturation of protein -TC compounds. From the safety and toxicological point of view, these findings show an additional advantage of cooking as a food processing method.

Cooking methods generally, can reduce the concentration of OTC in meat, boiling was more effective in reducing the concentration of OTC in muscle while roasting was more effective in reducing the concentration of liver samples**.** Since some of the reductions in OTC concentrations were not statistically significant, there is a possibility that their OTC concentrations after cooking, were still above the MRL. It is, therefore, better to prevent the occurrence of violative levels of drug residues in raw meats but where inevitable, cooking for a longer time should be explored.

## Authors’ Contributions

EEV planned, designed and carried out the work; OOJ was part of carrying out the work by raising the birds and involved in drug administration; NJA was part of the design and supervised the work. All authors read and approved the manuscript.
